# The learning curve of laparoscopic, robot-assisted and transanal total mesorectal excisions: a systematic review

**DOI:** 10.1007/s00464-022-09087-z

**Published:** 2022-06-13

**Authors:** Thijs A. Burghgraef, Daan J. Sikkenk, Paul M. Verheijen, Mostafa El Moumni, Roel Hompes, Esther C. J. Consten

**Affiliations:** 1 Department of Surgery, Meander Medical Center, Maatweg 3, 3813 TZ Amersfoort, the Netherlands; 2 Department of Surgery, University of Groningen, University Medical Center Groningen, Hanzeplein 1, 9713 GZ Groningen, the Netherlands; 3 Department of Surgery, University Medical Center Amsterdam, Location AMC, Amsterdam, the Netherlands

**Keywords:** Total mesorectal excision, Laparoscopy, Robot-assisted, Transanal, Learning curve

## Abstract

**Background:**

The standard treatment of rectal carcinoma is surgical resection according to the total mesorectal excision principle, either by open, laparoscopic, robot-assisted or transanal technique. No clear consensus exists regarding the length of the learning curve for the minimal invasive techniques. This systematic review aims to provide an overview of the current literature regarding the learning curve of minimal invasive TME.

**Methods:**

A systematic literature search was performed. PubMed, Embase and Cochrane Library were searched for studies with the primary or secondary aim to assess the learning curve of either laparoscopic, robot-assisted or transanal TME for rectal cancer. The primary outcome was length of the learning curve per minimal invasive technique. Descriptive statistics were used to present results and the MINORS tool was used to assess risk of bias.

**Results:**

45 studies, with 7562 patients, were included in this systematic review. Length of the learning curve based on intraoperative complications, postoperative complications, pathological outcomes, or a composite endpoint using a risk-adjusted CUSUM analysis was 50 procedures for the laparoscopic technique, 32–75 procedures for the robot-assisted technique and 36–54 procedures for the transanal technique. Due to the low quality of studies and a high level of heterogeneity a meta-analysis could not be performed. Heterogeneity was caused by patient-related factors, surgeon-related factors and differences in statistical methods.

**Conclusion:**

Current high-quality literature regarding length of the learning curve of minimal invasive TME techniques is scarce. Available literature suggests equal lengths of the learning curves of laparoscopic, robot-assisted and transanal TME. Well-designed studies, using adequate statistical methods are required to properly assess the learning curve, while taking into account patient-related and surgeon-related factors.

The cornerstone of therapeutic management of rectal cancer is surgical resection by total mesorectal excision (TME). This can be performed using several surgical approaches: open, laparoscopic (L-TME), robot-assisted (R-TME) and transanal TME (TaTME) [1–4]. Whereas the first TME was performed using open surgery, minimal invasive approaches are increasingly used since the introduction of laparoscopic rectal resections in the mid 90’s. The R-TME and TaTME technique were introduced in the beginning of the 00’s and 10’s respectively, in order to overcome technical limitations of the L-TME procedure.

With the introduction and implementation of a new surgical approach, surgeons need to climb a learning curve. This is the amount of procedures required to achieve an adequate surgical performance, regarding safety, efficacy and efficiency [5]. The ideal minimal invasive procedure has a short learning curve, and is therefore easy to master. In addition, the period in which the surgeon ‘climbs’ the learning curve should not result in additional morbidity, worsened oncological outcomes or mortality for the patient [6, 7].

It is suggested that L-TME and TaTME have a relatively long learning curve of around 50–90 procedures per surgeon [8–12], while R-TME is suggested to have a shorter learning curve [13–15]. Despite the number of papers reporting on learning curves of these approaches, the quality of evidence is limited. Patient populations are heterogeneous by including both benign and malignant diseases. Experience with previous techniques is mostly not taken into account, and some studies do not make a clear distinction between colonic and rectal resections. Additionally, multiple designs and statistical methods are used to assess the learning curve. Finally, although systematic reviews are available, some are outdated, or not restricted to rectal cancer surgery, while others do not evaluate the learning curve of all three minimal invasive techniques [16–20].

The aim of this systematic review is two-fold: First, we aim to create an overview of the current available literature regarding the learning curve of L-TME, R-TME and TaTME for patients with rectal carcinoma. Second, we aim to explore the impact of the learning curve on clinical outcomes in L-TME, R-TME and TaTME.

## Materials and methods

This systematic review was conducted and reported according to the PRISMA 2020 statement [21]. Approval of the Institutional Review Board (IRB) was deemed unnecessary, due to the nature of the study. Inclusion and exclusion criteria, as well as search strategies, the used critical appraisal tool, and outcomes of interest were prespecified. We did not register a review protocol in advance.

### Eligibility criteria

In order to create an overview of studies regarding the learning curve of L-TME, R-TME, and TaTME, studies were deemed eligible if: (1) the studies included patients with primary rectal cancer, or patients with colorectal cancer in which rectal cancer patients could be distinguished, (2) the patients underwent a TME, (3) the primary or secondary aim of the paper was to obtain the learning curve of either L-TME, R-TME or TaTME. Studies were excluded if they: (1) were written in other languages than English, German, French or Dutch, or if the studies (2) did not resemble an original article.

### Literature search and study selection

Two researchers independently conducted a systematic search (TAB and DJS) in PubMed, Embase and Cochrane Library on August 10, 2021. The following search terms were used: (rectum cancer OR colorectal cancer OR rectal OR colorectal) AND (learning curve OR learning), without limiting the search (for example to year of publication). After undoubling, title and abstract of all studies were screened for inclusion, and full text reading of the remaining studies was performed by two researchers independently. Finally, the reference lists of included studies were screened for possible eligible studies. Systematic reviews emerging in the literature search were excluded, but reference lists were screened for possible eligible studies. Disagreement between the two independent researchers was resolved through discussion until consensus was reached.

### Data collection

The primary outcome was length of the learning curve for L-TME, R-TME, and TaTME. Secondary outcomes included intraoperative, postoperative and oncological outcomes of patients operated during the learning curve, compared with patients operated after completion of the learning curve. In addition, statistical methods used to obtain the learning curve, as well as the outcome variables used to obtain the learning curve were recorded. A prespecified form was used to capture data of studies. This form contained the following data: author, year, country, study design, surgical technique, number of participating centers and surgeons, number of patients included, exclusion criteria and aim of the study. Additionally, surgeon-based or institute-based learning curve analysis, prior experience with the surgical technique, length of the learning curve based on intraoperative complications, length of the learning curve based on postoperative complications, length of the learning curve based on positive pathological circumferential margin (CRM), length of the learning curve based on operative time, length of the learning curve based on other variables or a compound variable, and used statistical methods for learning curve analysis were registered. Finally, if a comparison was performed between patients operated during the learning curve and after the learning curve was achieved, the following outcomes were compared: intraoperative complications, postoperative complications, positive CRM rate and operative time. All data was extracted by two researchers independently and disagreement was resolved through discussion.

### Outcomes

Length of the learning curve was specified as the number of procedures necessary to reach proficiency as identified by the specific study. Since studies used different clinical outcomes and statistical methods to assess proficiency of the surgical technique, length of the learning curve was reported per clinical outcome and statistical method used. Used clinical outcomes were: intraoperative complications; postoperative complications within 30 days; positive CRM rate, defined as a margin ≤ 1 mm; operative time, defined as time from incision to skin closure, or a composite of multiple clinical outcomes (i.e., conversion, local recurrence and postoperative complications). We registered length of the learning curve for each specific statistical method, and for CUSUM or RA-CUSUM analyses we differentiated between length of the learning curve based on deflection of the graph and stabilization of the graph, as the point at which the learning curve was achieved. Furthermore, a final conclusion per technique regarding length of the learning curve was defined as the reported lengths of the learning curve per technique as estimated only by RA-CUSUM analyses.

### Risk of bias

The MINORS tool [22] was used to assess the quality of the studies. Both researchers (TAB and DJS) recorded the data independently. Disagreement was resolved through discussion until consensus was reached.

## Results

### Study selection

**Fig. 1:**
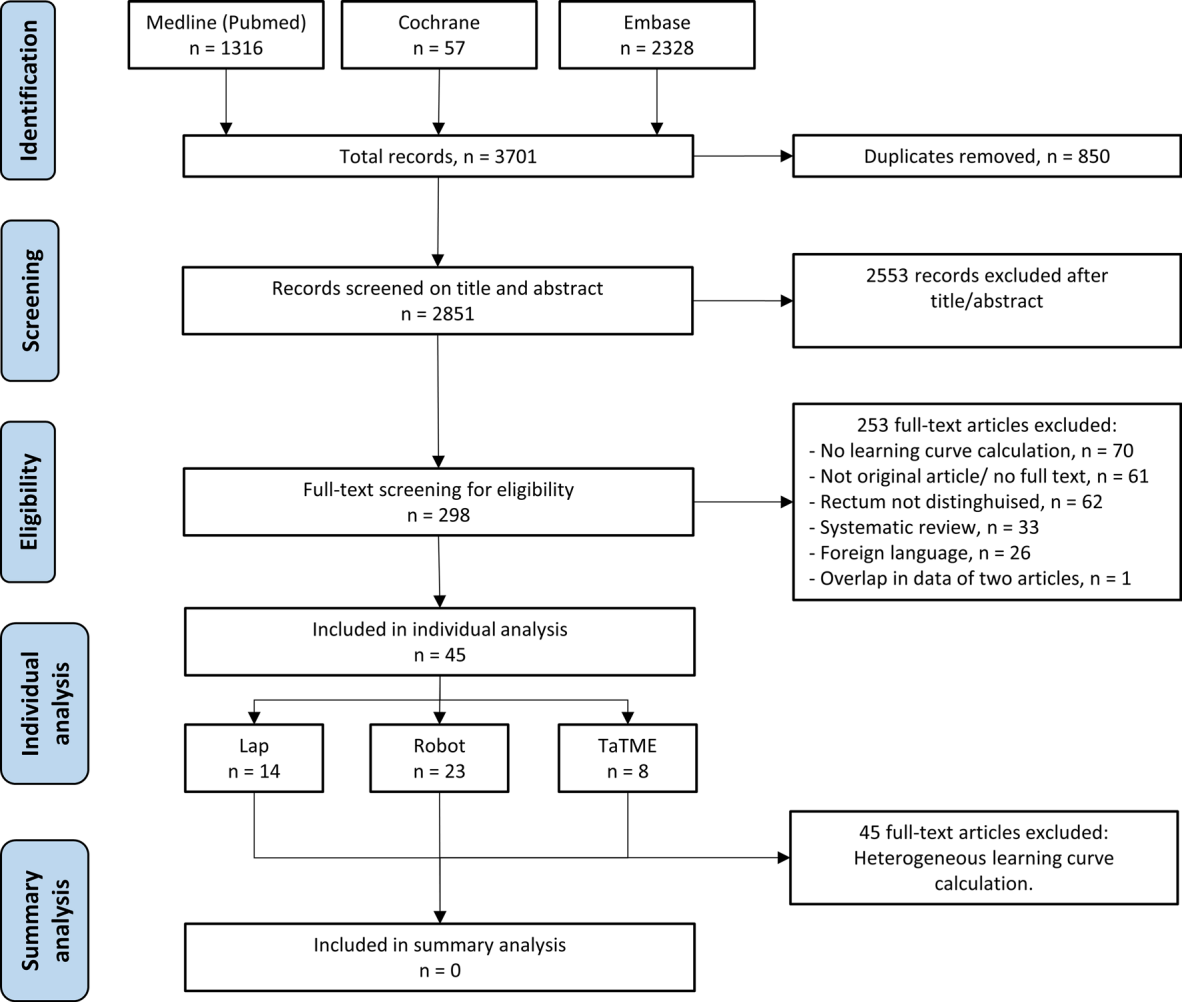
Flow diagram of study selection. *Lap* Studies involving laparoscopic total mesorectal excision, *Robot* Studies involving robot-assisted total mesorectal excision, *TaTME* Studies involving transanal total mesorectal excision

PubMed, Embase and Cochrane Library were searched on August 10, 2021 and yielded 3701 records. After undoubling 2851 records remained. Screening title and abstract for eligibility resulted in 298 records. After full text screening, an additional 253 records were excluded. This resulted in 45 records that were included in this systematic review. Studies were too heterogeneous, both clinically and methodologically, to perform a meta-analysis (Fig. 1).

### Study characteristics

The characteristics of included studies are presented in Table 1. Studies were published between 2009 and 2021, with a total of six prospective studies [11, 23–27], 34 retrospective studies [9, 10, 12, 14, 15, 28–56], and five studies in which the design was not clearly described [8, 13, 57–59]. Thirteen studies reported on the learning curve of L-TME [10–12, 26, 27, 39–44, 58, 59], twenty on the learning curve of R-TME [13–15, 23–25, 28–35, 52–57], eight on the learning curve of TaTME [8, 9, 36–38, 48–50], and four reported on the comparison of the learning curve of two approaches [45–47, 51].

**Table 1:** Study characteristics of included studies

Author, year	Country	Study design	Technique	Centers	Surgeons	Patients	Exclusion criteria	Learning curve study aim
Kim (2014) [14]	South Korea	Retrospective	R-TME	1	1	167	None	Primary aim
Akmal (2012) [23]	South Korea	Prospective	R-TME	1	1	80	None	Primary aim
Foo (2015) [24]	Hong Kong	Prospective	R-TME	1	1	39	Abdominoperineal resection, Hartmann resection	Primary aim
Sng (2013) [52]	South Korea	Retrospective	R-TME	1	1	197	Low rectal tumor, > 5 cm size Male, T4b, anterior invasion	Primary aim
Jiménez-Rodriguez (2013) [13]	Spain	Not mentioned	R-TME	1	3	43	None	Primary aim
Yamaguchi (2015) [53]	Japan	Retrospective	R-TME	1	1	80	None	Primary aim
Kim (2014) [54]	South Korea	Retrospective	R-TME	1	2	200	None	Primary aim
Odermatt (2017) [55]	United Kingdom	Retrospective	R-TME	1	2	90	None	Primary aim
Kawai (2018) [56]	Japan	Retrospective	R-TME	1	1	131	None	Primary aim
Park (2014) [15]	South Korea	Retrospective	R-TME	1	1	130	Synchronous procedure Lateral lymph node dissection	Primary aim
Byrn (2014) [28]	United States	Retrospective	R-TME	1	1	51	History of laparotomy for abdominopelvic surgery Large risk of conversion, extreme age or comorbidities	Primary aim
Morelli (2016) [29]	Italy	Retrospective	R-TME	1	1	50	None	Secondary aim
Kim (2012) [25]	South Korea	Prospective	R-TME	1	1	62	Acute surgery, acute obstruction History of abdominal surgery, severe cardiopulmonary disease	Primary aim
Kuo (2014) [30]	Taiwan	Retrospective	R-TME	1	1	36	None	Secondary aim
D’Annibale (2013) [31]	Italy	Retrospective	R-TME	1	1	50	None	Secondary aim
Lee (2020) [35]	South Korea	Retrospective	R-TME	1	1	506	No adenocarcinoma, palliative intent	Primary aim
Olthof (2020) [32]	The Netherlands	Retrospective	R-TME	1	2	100	None	Primary aim
Aghayeva (2020) [33]	Turkey	Retrospective	R-TME	1	unclear	96	Abdominoperineal resection Missing value for operative time	Primary aim
Gachabayov (2020) [57]	USA, South Korea, Spain, Taiwan, Italy, Russia	Not mentioned	R-TME	5	5	235	None	Primary aim
Noh (2020) [34]	South Korea	Retrospective	R-TME	1	5	662	Abdominoperineal resection, other synchronous surgical procedures Palliative intent, R2 resection for macroscopic residual disease	Primary aim
Koedam (2018) [8]	The Netherlands	Not mentioned	TaTME	1	3	138	None	Primary aim
Lee (2018) [9]	United States	Retrospective	TaTME	1	4	87	High rectum carcinoma Benign lesions or lesions fit for local excision	Primary aim
Mege (2018) [36]	France	Retrospective	TaTME	1	1	34	Tumor in mid or high rectum, Abdominoperineal resection	Primary aim
Rubinkiewicz (2020) [37]	Poland	Retrospective	TaTME	1	1	66	None	Primary aim
Persiani,2020[38]	Italy	Retrospective	TaTME	1	1	121	TaTME for IBD or locoregional recurrence after previous rectal surgery High rectal cancer	Primary aim
Caycedo-Marulanda (2020) [48]	Canada	Retrospective	TaTME	1	1	100	High rectal cancer	Primary aim
Zeng (2021) [50]	China	Retrospective	TaTME	1	1	171	T4b, stage IV tumors, emergency surgery	Primary aim
Oostendorp, 2021 [49]	The Netherlands	Retrospective	TaTME	6	Unclear	624	None	Primary aim
Balik (2010) [39]	Turkey	Retrospective	L-TME	1	3	284	Emergency surgery, inoperability	Primary aim
Tsai (2015) [40]	Taiwan	Retrospective	L-TME	1	1	39	Abdominoperineal resection, Hartmann resection Conversion and single port laparoscopy	Primary aim
Bege (2010) [11]	France	Prospective	L-TME	1	1	127	T4 or fixed tumor, synchronous liver resection Abdominoperineal resection Medical contraindication or refusal for laparoscopy	Primary aim
Lujan (2014) [41]	Spain	Retrospective	L-TME	1	2	120	BMI > 35, carcinoma in lower 1/3 of the rectum	Primary aim
Kayano (2011) [58]	Japan	Not mentioned	L-TME	1	1	250	Combined resections (cholecystectomy, hepatectomy, hysterectomy)	Primary aim
Agha, 2008[42]	Germany	Retrospective	L-TME	1	6	300	Acute resection, transanal local resections Local recurrent disease	Secondary aim
Ito (2009) [59]	Japan	Not mentioned	L-TME	1	Multiple	200	T3-T4 tumor, T2 carcinoma in middle or lower rectum	Secondary aim
Son (2010) [12]	South Korea	Retrospective	L-TME	1	1	431	Inoperable disease	Primary aim
Fukunaga (2008) [26]	Japan	Prospective	L-TME	1	1	97	Emergency resection, abdominoperineal resection, obstruction Morbid obesity, prior major lower abdominal surgery Tumor occupying most of the pelvis, carcinoma below peritoneal deflection Lateral lymph node dissection,	Secondary aim
Kim (2014) [10]	South Korea	Retrospective	L-TME	1	1	512	Palliative resection, Abdominoperineal resection, Hartmann resection	Primary aim
Park (2009) [27]	South Korea	Prospective	L-TME	1	1	Unknown	None	Secondary aim
Kuo (2013) [43]	Taiwan	Retrospective	L-TME	1	2	28	Low anterior resection without need for intersphincteric resection	Secondary aim
Wu (2017) [44]	China	Retrospective	L-TME	1	3	281	ASA 4, BMI > 35, Neoadjuvant therapy, pregnancy History of major abdominal surgery, malignancy within 5 years Metastatic or in situ disease, palliative resection, emergency resection	Primary aim
Melich (2015) [45]	South Korea	Retrospective	R-TME vs L-TME	1	1	92 vs 106	Combined procedure	Primary aim
Morelli (2018) [46]	Italy	Retrospective	R-TME Si vs R-TME Xi	1	1	40 vs 40	None	Secondary aim
Park (2014) [47]	South Korea	Retrospective	R-TME vs L-TME	1	1	89 vs 89	Synchronous operation Lateral lymph node dissection	Primary aim
Wang (2021) [51]	China	Retrospective	R-TME vs L-TME	1	1	40 vs 65	Combined resections, palliative resections, ASA IV, previous abdominal pelvic surgery	Primary aim

*TME* Total mesorectal excision, *L-TME* Laparoscopic TME, *R-TME* Robot-assisted TME, *TaTME* Transanal TME, *ASA* American Society of Anesthesiology classification, *BMI* Body mass index

In total 7562 patients were included in this systematic review. The average number of included patients was 150 for R-TME studies, 168 for TaTME studies and 205 for L-TME studies. Most studies’ primary aim was to define the learning curve, though for nine studies it was a secondary aim [26, 27, 29–31, 42, 43, 46, 59]. Thirteen studies reported on institutional learning curves [8, 9, 11, 13, 31–33, 39, 41–43, 49, 59], while the others reported on surgeons’ individual learning curves. Previous experience with colorectal surgery was mentioned in twenty-one studies [8–10, 13, 15, 24, 25, 28, 29, 31, 35, 37, 39–42, 45–47, 51–56]. The majority of studies defined exclusion criteria, while seventeen did not exclude patients during the learning curve [8, 13, 14, 23, 27, 29–32, 37, 46, 48, 49, 53–57].

### Risk of bias

None of the studies scored high on all criteria of the MINORS tool. Nineteen out of 41 non-comparative studies adequately reported more than half of the required criteria [9–12, 15, 24, 25, 27, 33, 35, 37, 38, 48–50, 53, 55]. Study quality was highest among the TaTME studies, and varied most among the R-TME and L-TME studies. All comparative studies adequately reported more than half of the MINORS criteria [45–47]. One study prospectively calculated the study size [9] and seventeen used adequate statistical analyses [8–10, 12–14, 24, 35, 37, 38, 44, 45, 47, 50, 52, 55]. Regarding the use of adequate definitions of clinical outcome variables, nineteen studies adequately reported unbiased assessment of endpoints [8, 10, 15, 24, 25, 27, 33, 35, 38, 39, 47–57, 60] (Table 2).

**Table 2:** Risk of bias assessment according to MINORS tool

Author/year	Clearly stated aim	Inclusion of consecutive patients	Prospective collection of data	Endpoints appropriate to the aim	Unbiased assessment of endpoints	FU appropriate for study aim	Loss to follow up < 5%	Prospective calculation of the study size	Adequate control group	Contemporary groups	Baseline equivalence of groups	Adequate statistical analyses
Kim (2014) [14]	2	0	1	2	1	0	0	0	NA	NA	NA	2
Akmal (2012) [23]	2	2	2	1	0	NA	0	0	NA	NA	NA	1
Foo (2015) [24]	2	1	2	1	2	NA	0	0	NA	NA	NA	2
Sng (2013) [52]	2	1	1	1	2	NA	0	0	NA	NA	NA	2
Jiménez-Rodriguez (2013) [13]	2	2	0	2	1	0	0	0	NA	NA	NA	2
Yamaguchi (2015) [53]	2	2	1	1	2	NA	0	0	NA	NA	NA	2
Kim (2014) [54]	2	2	1	1	2	NA	0	0	NA	NA	NA	2
Odermatt (2017) [55]	2	2	1	2	2	2	0	0	NA	NA	NA	2
Kawai (2018) [56]	2	2	1	1	2	NA	0	0	NA	NA	NA	2
Park (2014) [15]	2	2	1	2	2	1	0	0	NA	NA	NA	2
Byrn (2014) [28]	1	1	1	1	0	NA	0	0	NA	NA	NA	1
Morelli (2016) [29]	2	2	1	1	1	NA	0	0	NA	NA	NA	2
Kim (2012) [25]	2	2	2	1	2	NA	0	0	NA	NA	NA	1
Kuo (2014) [30]	2	0	1	1	1	NA	0	0	NA	NA	NA	1
D’Annibale (2013) [31]	2	2	1	1	1	NA	0	0	NA	NA	NA	1
Lee (2020) [35]	2	2	1	2	2	2	0	0	NA	NA	NA	2
Olthof (2020) [32]	2	2	1	2	2	NA	0	0	NA	NA	NA	2
Aghayeva (2020) [33]	2	2	1	1	2	NA	0	0	NA	NA	NA	2
Gachabayov (2020) [57]	2	2	0	1	2	NA	0	0	NA	NA	NA	2
Noh (2020) [34]	2	2	1	2	2	2	0	0	NA	NA	NA	2
Koedam (2018) [8]	2	2	0	2	2	2	0	0	NA	NA	NA	2
Lee (2018) [9]	2	2	1	2	1	2	0	2	NA	NA	NA	2
Caycedo-Marulanda (2020) [48]	2	2	1	1	2	2	0	0	NA	NA	NA	2
Mege (2018) [36]	2	2	1	1	1	NA	0	0	NA	NA	NA	1
Rubinkiewicz (2020) [37]	2	2	1	2	1	NA	0	0	NA	NA	NA	2
Persiani (2020) [38]	2	2	2	2	2	2	0	0	NA	NA	NA	2
Zeng (2021) [50]	2	2	1	1	2	NA	0	0	NA	NA	NA	2
Oostendorp (2021) [49]	2	2	1	2	2	2	0	0	NA	NA	NA	1
Balik (2010) [39]	2	0	1	1	2	1	0	0	NA	NA	NA	1
Tsai (2015) [40]	2	1	1	1	1	NA	0	0	NA	NA	NA	1
Bege (2010) [11]	1	1	2	2	1	1	0	0	NA	NA	NA	2
Lujan (2014) [41]	2	1	1	1	1	2	0	0	NA	NA	NA	1
Kayano (2011) [58]	2	2	0	1	1	0	0	0	NA	NA	NA	1
Agha (2008) [42]	1	0	1	1	1	NA	0	0	NA	NA	NA	1
Ito (2009) [59]	2	0	0	1	0	0	0	0	NA	NA	NA	1
Son (2010) [12]	2	2	1	2	1	0	0	0	NA	NA	NA	2
Fukunaga (2008) [26]	1	1	2	1	1	NA	0	0	NA	NA	NA	1
Kim (2014) [10]	2	1	0	2	2	2	2	0	NA	NA	NA	2
Park (2009) [27]	1	0	2	2	2	2	0	0	NA	NA	NA	1
Kuo (2013) [43]	2	0	1	1	0	1	0	0	NA	NA	NA	1
Wu (2017) [44]	2	1	1	1	1	NA	0	0	NA	NA	NA	2
Melich (2015) [45]	2	2	1	2	1	0	0	0	2	2	1	2
Morelli (2018) [46]	2	2	1	1	1	NA	0	0	2	1	1	1
Park (2014) [47]	2	1	1	1	2	NA	0	0	2	2	1	2
Wang (2021) [51]	2	1	1	1	2	NA	0	0	2	2	2	1

*NA* not applicable (Assessment score: 2 = adequately reported, 1 = inadequately reported, 0 = not reported)

#### Statistical methods of learning curve analyses

Most studies used a combination of different learning curve analyses. No clear learning curve analysis was used in three studies [23, 36, 54], eleven studies used split group analyses (SGA) or sequence analysis for one or more clinical outcome variables [12, 25–28, 39, 41–43, 49, 58, 59] and twelve studies used the moving average analysis (MAA) [10, 12, 14, 30, 40, 44, 45, 47, 58, 60]. Eighteen studies used the CUSUM analysis based on operative time [8, 9, 13, 15, 24, 29, 31–34, 37, 44, 46, 47, 50–53, 55–57]. Two studies used the CUSUM analysis based on intraoperative complications [12, 37], six studies used the CUSUM analysis based on postoperative complications [11, 12, 32, 37, 45, 48], one study based the CUSUM analysis on positive CRM rate [45] and seven studies used the CUSUM analysis based on a composite outcome [9, 11, 13–15, 34, 35].

One or more risk-adjusted CUSUM analyses (RA-CUSUM) were used in eight studies: three studies used postoperative morbidity [8, 9, 35, 38], two studies used positive CRM rate [10, 35], one study used local recurrence [10]. Another study used conversion [12] and three studies used a composite outcome [14, 15, 35]. Finally, some studies used the first deflection in the (RA-)CUSUM or MAA graph as the point at which proficiency was reached [8, 10, 12, 14, 15, 27, 29, 31, 33, 34, 44, 46, 47, 52, 53, 55–57], while others defined proficiency as the point at which stabilization was reached [9, 13, 24, 30, 35, 37, 38, 40, 58] (Tables 3 and 4).

**Table 3:** Results of individual studies regarding statistical analysis and learning curve

Author, year	Technique	Learning curve characteristics		Learning curve analysis										Conclusion according to article	
		Analysis	Previous experience with surgical technique	Variable (IOC)	Analysis	Variable (POC)	Analysis	CRM rate	Analysis	Operative time	Analysis	Other variable	Analysis	Length	
Kim (2014) [14]	R-TME	Per surgeon	Not mentioned	–	–	–	–	–	–	Operative time Console time	MAA_D_: 33 MAA_S_: 72	Combination: Conversion, IOC, POC, CRM + , OT > 2 SD	RA-CUSUM_D_: 32	32	
Akmal (2012) [23]	R-TME	Per surgeon	Not mentioned	–	–	–	–	–	–	–		–	–	–	
Foo (2015) [24]	R-TME	Per surgeon	30 robotic RR assisted < 5 open/lap RR	–	–	–	–	–	–	Operative time	CUSUM_D_: 8 CUSUM_S_: 25	–	–	25	
Sng (2013) [52]	R-TME	Per surgeon	> 2000 lap CRR > 1000 lap RR	–	–	–	–	–	–	Console time	CUSUM_D_: 35 CUSUM_S_: 128	–	–	35	
Jiménez-Rodriguez (2013) [13]	R-TME	Per institute	Long experience in lap Robot training	–	–	–	–	–	–	Operative time	CUSUM_D_: 11 CUSUM_S_: 21	Combination: Conversion, IOC, POC, mortality	CUSUM_D_: 11 CUSUM_S_: 23	23	
Yamaguchi (2015) [53]	R-TME	Per surgeon	Expert in RR	–	–	–	–	–	–	Operative time	CUSUM_D_: 25 CUSUM_S_: 50	–	–	25	
Kim (2014) [54]	R-TME	Per surgeon	Surg A 200 open CRR, < 30 lap Surg B 800 open CRR, > 300 lap Robot training	–	–	–	–	–	–	Operative time	–	–	–	–	
Odermatt (2017) [55]	R-TME	Per surgeon	Surg A: 1500 CRR Surg B: 400 CRR Robot training	–	–	Morbidity (CD 3b-5)	–	–	–	Operative time	CUSUM_D_: 7 (Surg A) CUSUM_D_: 15 (Surg B)	–	–	15	
Kawai (2018) [56]	R-TME	Per surgeon	Substantial lap CRR Robot training	–		–		–		Console time	CUSUM_D_: 19 CUSUM_S_: 78	–	–	19	
Park (2014) [15]	R-TME	Per surgeon	2 year CRC fellowship 6 lap, 10 open CRR	–	–	–	–	–	–	Operative time	CUSUM_D_: 44 CUSUM_S_: 78	Combination: Conversion, R1, < 12 LN, LR, POC	RA-CUSUM_D_: 75	75	
Byrn (2014) [28]	R-TME	Per surgeon	1 year staff level experience of lap pelvic dissection	–	–	–	–	–	–	Operative time	SGA: -	–	–	–	
Morelli (2016) [29]	R-TME	Per surgeon	> 500 lap procedures	–	–	–	–	–	–	Operative time	CUSUM_D_: 19	–	–	19	
Kim (2012) [25]	R-TME	Per surgeon	> 20 year experience in open RR	–	–	–	–	–	–	Operative time	SGA: 20	–	–	20	
Kuo (2014) [30]	R-TME	Per surgeon	Not mentioned	–	–	–	–	–	–	Operative time	MAA: 19	–	–	19	
D’Annibale (2013) [31]	R-TME	Per institute	Not mentioned	–	–	–	–	–	–	Operative time	Sequence: 25 CUSUM_D_: 22	–	–	–	
Lee (2020) [35]	R-TME	Per surgeon	3000 lap TMEs	–	–	Morbidity (CD 3–5)	RA-CUSUM_S_: 191	CRM + DRM +	RA-CUSUM_S_: 418	–	–	Combination: Conversion, CD 3–5, R1, < 12 LN or < 8 LN (CRT)	RA-CUSUM_D_: 177	177	
Olthof (2020) [32]	R-TME	Per Institute	Intuitive training program Experienced colorectal center	–	–	CCI Major morbidity (CD 3–5)	CUSUM: 40 CUSUM: 40			Operative time	CUSUM: 20	Anastomotic leakage	CUSUM: 30–40	40	
Aghayeva (2020) [33]	R-TME	Per institute	Not mentioned	–	–	–	–	–	–	Operative time	CUSUM_D_: 52	–	–		52
Gachabayov (2020) [57]	R-TME	Per surgeon	Not mentioned	–	–	–	–	–	–	Operative time	CUSUM_D_: 8–25 CUSUM_S_: 12–56	–	–		–
Noh (2020) [34]	R-TME	Per surgeon	Not mentioned Different previous lap experience	–	–	–	–	–	–	Operative time	CUSUM_D_: 23–110	Local failure (CRM + , LR) Surgical failure (conversion, AL)	CUSUM_D_: - CUSUM_D_: -		23–110
Koedam (2018) [8]	TaTME	Per Institute	> 75 lap CRR resect annual > 30 TAMIS annual	–	–	Morbidity (CD 3b-5)	RA-CUSUM_D_: 40	–	–	Operative time	CUSUM_D_: 80	–	–		40
Lee (2018) [9]	TaTME	Per institute	Proficient in lap RR Proficient in TAMIS	-	-	Morbidity	RA-CUSUM_D_:29 RA-CUSUM_S_: 36		-	Operative time	CUSUM_D_: 36 CUSUM_S_: 51	Combination: R1, incomplete TME quality,	CUSUM_D_: 36 CUSUM_S_: 51		51
Caycedo-Marulanda (2020) [48]	TaTME	Per institute	Not mentioned	–	–	–	–	–	–	–	–	Anastomotic leakage	CUSUM: 50		45–51
Mege (2018) [36]	TaTME	Per surgeon	Not mentioned	–	–	–	–	–	–	–	–	–	–		–
Rubinkiewicz (2020) [37]	TaTME	Per surgeon	Training in reference centers	Yes	CUSUM_S_: 40	Morbidity	CUSUM_S_:30	–	–	Operative time	CUSUM_S_: 40	TME quality	CUSUM_S_: -		40
Persiani (2020) [38]	TaTME	Per Institute	Not mentioned	–	–	Morbidity	RA- CUSUM_D_:24 RA- CUSUM_S_:69 Bernoulli CUSUM: 21 Reference CUSUM:108			Operative time	RA- CUSUM_D_:54 RA- CUSUM_S_:87 Bernoulli CUSUM: 71	Reoperation rate	RA- CUSUM_D_:54 RA- CUSUM_S_:54 Bernoulli CUSUM: 31 Reference CUSUM: 69		71
						Major Morbidity	RA- CUSUM_D_:54 RA- CUSUM_S_:54 Bernoulli CUSUM: 55 Reference CUSUM: 54					Anastomotic leakage	RA- CUSUM_D_:78 RA- CUSUM_S_:78 Bernoulli CUSUM: 42 Reference CUSUM: 42		
Zeng (2021) [50]	TaTME	Per surgeon	Not mentioned	–	–	–	–	–	–	Operative time	CUSUM_D_: 42 CUSUM_S_: 95	–	–		42–95
Oostendorp (2021) [49]	TaTME	Per Institute	Not mentioned	–	–	–	–	–	–	–	–	Local recurrence	SGA: -		–

*POC* Postoperative complication, *IOC* Intraoperative complication, *RR* Rectal resection, *CRR* Colorectal resection, *CR* Colon resection, *R-TME* robot-assisted TME, *TaTME* transanal TME, *TAMIS* Transanal minimal invasive surgery, *CUSUM* Cumulative sum analysis, *RA-CUSUM* Risk-adjusted cumulative sum analysis, *CUSUMD* Deflection point in CUSUM, *CUSUMS* Stabilization point in CUSUM, *MAA* Moving average analysis, *MAAS* Moving average stabilization point, *MAAD* Moving average deflection point, *SGA* Split group analysis, *CD* Clavien–Dindo classification, *CRM* Circumferential resection margin, *LN* Lymph nodes, *AL* Anastomotic leakage, *LR* Local recurrence, *AL* Anastomotic leakage, *SSI* Surgical site infection, *LR* Local recurrence, *CRT* Neoadjuvant chemoradiotherapy, *CCI* Comprehensive complication index

**Table 4:** Results of individual studies regarding statistical analysis and learning curve

Author, year	Technique	Learning curve characteristics		Learning curve analysis										Conclusion according to article
		Analysis	Previous experience with surgical technique	Variable (IOC)	Analysis	Variable (POC)	Analysis	CRM rate	Analysis	Operative time	Analysis	Other variable	Analysis	Length
Balik (2010) [39]	L-TME	Per institute	305 open + lap CR	–	–	–	–	–	–	Operative time	SGA: - Sequence: -	–	–	–
Tsai (2015) [40]	L-TME	Per surgeon	Fellowship completed Little experience in lap	–	–	–	–	–	–	Operative time	MAA: 22	–	–	22
Bege (2010) [11]	L-TME	Per Institute	Not mentioned	–	–	Morbidity	CUSUM_D_:45	–	–	–	–	Combination (comb): POC, LR, Conversion, R1	CUSUM_D_: 50	50
Lujan (2014) [41]	L-TME	Per institute	Ample experience in open CRR Skilled advanced lap	–	–	–	–	–	–	Operative time	Sequence: - SGA: -	–	–	–
Kayano (2011) [58]	L-TME	Per surgeon	Not mentioned		–	Morbidity	SGA: 200	–	–	Operative time	MAA: 50	Conversion	SGA: 150	–
Agha (2008) [42]	L-TME	Per institute	Experience with lap CR No experience with lap RR	–	–	SSI	SGA: 20	–	–	Operative time	SGA: 40	–	–	–
Ito (2009) [59]	L-TME	Per institute	Not mentioned	–	–	Morbidity	SGA: -	–	–	Operative time	SGA: 40	–	–	–
Son (2010) [12]	L-TME	Per surgeon	Not mentioned	Yes	CUSUM_D_: 243	Morbidity	CUSUM_D_: 79	–	–	Operative time	MAA:61	Conversion Transfusion volume	RA-CUSUM_D_l: 61 (Conv) SGA:75 (Transfusion volume)	79
Fukunaga (2008) [26]	L-TME	Per surgeon	Not mentioned	–	–	–	–	–		Operative time	Sequence: -	–	–	–
Kim (2014) [10]	L-TME	Per surgeon	A: Fast experience B: Trained by A	–	–	–	–	CRM +	RA-CUSUM_D_:50 (A) RA-CUSUM_D_:70 (B)	Operative time	MAA_A_: 90 MAA_B_: 90	LR	RA- CUSUM_D_:110 (A) RA- CUSUM_D_: 110 (B)	110
Park (2009) [27]	L-TME	Per surgeon	Not mentioned	–	–	–	–	–	–	Operative time	MAA: 30	LR Conversion	SGA: 69 (LR) CUSUM_D_: 13 (Conversion)	69
Kuo (2013) [43]	L-TME	Per institute	Not mentioned	–	–	–	–	–	–	Operative time	SGA: 17	–	–	17
Wu (2017) [44]	L-TME	Unclear	Not mentioned	–	–	–	–	–	–	Operative time	CUSUM_D_: 36–42 MAA: 36–47	–	–	40
Melich (2015) [45]	R-TME vs L-TME	Per surgeon	700 open CR, 50 open RR, 150 lap CR	–	–	AL, intra-abdominal abscess	CUSUM: -	CRM +	CUSUM: -	Operative time	MAA: -	–	–	–
Morelli (2018) [46]	R-TMESi vs R-TME Xi	Per surgeon	> 100 RR > 100 lap surgery	–	–	–	–	–	–	Operative time	CUSUM_D_: 19	–	–	19
Park (2014) [47]	R-TME vs L-TME	Per surgeon	2 year lap CRR fellowship	–	–	–	–	–	–	Operative time	CUSUM_D_: 44 (robot) MAA: 21 (robot) MAA: 69 (lap) CUSUM_D_: 41 (lap)	–	–	44 (robot) 41 (lap)
Wang (2021) [51]	R-TME vs L-TME	Per surgeon	> 300 open CRR > 150 lap CRR Robot training	–	–	–	–	–	–	Operative time	CUSUM_D_: 17 (rob) CUSUM_D_: 34 (lap)	–	–	17 (rob) 34 (lap)

*POC* Postoperative complication, *IOC* Intraoperative complication, *RR* Rectal resection, *CRR* Colorectal resection, *CR* Colon resection, *lap* Laparoscopy, *TAMIS* Transanal minimal invasive surgery, *CUSUM* Cumulative sum analysis, *RA-CUSUM* Risk-adjusted cumulative sum analysis, *CUSUMD* Deflection point in CUSUM, *CUSUMS* Stabilization point in CUSUM, *MAA* Moving average analysis, *MAAS* Moving average stabilization point, *MAAD* Moving average deflection point, *SGA* Split group analysis, *CD* Clavien–Dindo classification, *CRM* Circumferential resection margin, *LN* Lymph nodes, *AL* Anastomotic leakage, *LR* Local recurrence, *AL* Anastomotic leakage, *SSI* Surgical site infection, *LR* Local recurrence, *CRT* Neoadjuvant chemoradiotherapy, *R-TME* robot assisted TME, *L-TME* laparoscopic TME

#### Length of the learning curve

Despite the fact that all studies assessed the learning curve as their primary or secondary outcome, only 31 studies defined the number of procedures necessary to complete the learning curve based on their results [8, 9, 11, 13–15, 24, 25, 29, 30, 32, 34, 35, 37, 38, 40, 48, 50–53, 55, 56]. CUSUM analyses for length of the learning curve based on operative time differed between 19 and 128 for R-TME, between 51 and 95 for TaTME and between 36 and 42 for L-TME. The only study using RA-CUSUM for length of the learning curve based on operative time showed 87 procedures to be the learning curve for TaTME [38].

Length of the learning curve based on specific clinical outcomes differed widely. Two studies used intraoperative complications as the variable for the calculation of the learning curve: a TaTME study and a L-TME study estimated the learning curve to be respectively 40 and 243 patients using the CUSUM method [12, 37]. Additionally, two studies used positive CRM as oncological variable for the analyses of the learning curve, both using RA-CUSUM analyses: Length of the learning curve was 418 in a R-TME study [35] and 50–70 in a L-TME study [10]. Most studies calculated the learning curve based on postoperative morbidity: using CUSUM analyses lengths differed between 45 and 79 for L-TME studies [11, 12], 40–191 for R-TME studies [32, 35], and 21–108 for TaTME studies [8, 9, 37, 38]. When only taking into account RA-CUSUM analyses, lengths were 191 for R-TME [35] and between 24 and 54 for TaTME [8, 9, 38]. No RA-CUSUM analysis was conducted for L-TME.

Lengths of the learning curve using (RA-)CUSUM analyses based on compound outcome of clinical variables, were 11, 32, 75 and 177 for four R-TME studies and 36 for a TaTME study. No RA-CUSUM analysis was conducted for L-TME. A CUSUM analysis based on compound outcomes showed a length of 50 procedures in a L-TME study [9, 11, 35, 61]. When only taking into account RA-CUSUM analyses based on a compound outcome, length of the learning curve was between 32 and 177 for R-TME, 36 for TaTME, while this was not performed for L-TME.

Finally, taking into account all RA-CUSUM analyses of clinical outcomes only, length of the learning curve was between 50 and 70 for L-TME, 32–418 for R-TME and 36–54 for TaTME.

#### Before-after learning curve comparison

After establishing a learning curve, 23 studies reported on the comparison of outcomes between patients that had been operated during the learning curve and patients that had been operated after completing the learning curve [8, 9, 11, 13–15, 24, 25, 29, 30, 32, 35, 37, 38, 43, 46, 47, 51–53, 56–58]. Bege et al., who used postoperative complications to assess the learning curve, showed a decline in postoperative morbidity after the learning curve for L-TME was reached [11]. Rubinkiewicz et al., who used postoperative morbidity, intraoperative morbidity, operative time and a composite outcome to assess the learning curve of TaTME, showed a significant decline in postoperative morbidity and intraoperative morbidity after the learning curve was reached [37]. Operative times were significantly reduced in thirteen studies after the learning curve was reached [8, 14, 15, 24, 33, 37, 38, 46, 47, 51–53, 56, 57] (Table 5). Eight of these studies used operative time to assess the learning curve. While in three R-TME studies and two TaTME studies the learning curve was based on clinical outcomes [8, 14, 15, 35, 38].

**Table 5:** Comparison of outcomes during the learning curve and after the learning curve

Author, year	Comparison	Technique	During learning curve				After learning curve			
			Intraop complications	Postop complications	CRM +	Operative time	Intraop complications	Postop compicationsl	CRM +	Operative time
Kim (2014) [14]	32 vs 135	R-TME	3 (9.4%)	5 (15.6%)	3 (9.4%)	252 (42) *	7 (5.2%)	23 (17.0%)	5 (3.7%)	203 (46) *
Foo (2015) [24]	25 vs 14	R-TME	–	4 (16%)	0	446 (102) *	–	0	2 (14.3%)	311 (165) *
Sng (2013) [52]	35 vs 162	R-TME	–	6 (17.1%)	0	265 (190–470) *	–	68 (42.0%)	2 (1.2%)	270 (145–515) *
Jiménez-Rodriguez (2013) [13]	23 vs 20	R-TME	3 (13.0%)	5 (21.7%)	–	189 (39)	0	1 (5.0%)	–	208 (44)
Yamaguchi (2015) [53]	25 vs 55	R-TME	–	3 (12.0%)	–	415 (156–683) *	–	5 (9.1%)	–	240 (135–529) *
Kawai (2018) [56]	19 vs 111	R-TME	–	2 (11.8%)	–	305 (111) *	–	13 (11.7%)	–	227 (112) *
Park (2014) [15]	78 vs 52	R-TME	–	8 (10.3%)	6 (7.7%)	212 (110–338) *	–	15 (28.8%)	3 (5.8%)	182 (109–376) *
Morelli, 2016[29]	19 vs 31	R-TME	–	7 (35.0%)	–	–	–	9 (29.0%)	–	–
Kim (2012) [25]	20 vs 42	R-TME	–	3 (15.0%)	–	454 (112)	–	5 (11.9%)	–	359 (62)
Kuo (2014) [30]	19 vs 17	R-TME	–	–	1 (5.3%)	520 (360–720)	–	–	3 (17.6%)	448 (315–585)
Lee (2020) [35]	177 vs 329	R-TME	–	48 (27.1%)	10 (5.4%)	361 (313–432)	–	77 (23.5%	19 (5.9%)	337 (292–398)
Aghayeva (2020) [33]	52 vs 44	R-TME	–	15 (28.8%)	2 (3.9%)	380 (109)*	–	7 (15.9%)	1 (2.7%)	323 (103)*
Gachabayov (2020) [57]	83 vs 152	R-TME	–	20 (24.1%)	–	244 (123)*	–	51 (33.5%)	–	192 (100) *
Koedam (2018) [8]	40 vs 98	TaTME	–	23 (57.5%)	1 (2.5%)	199 (95–329) *	–	53 (54.1%)	1 (1.0%)	153 (80–261) *
Lee (2018) [9]	51 vs 36	TaTME	6 (12%)	23 (45%)	2 (4%)	278 (84)	2 (6%)	15 (42%)	0	270 (73)
Rubinkiewicz (2020) [37]	40 vs 26	TaTME	5 (20%) *	13 (33%) *	–	270 (240–300) *	1 (13%) *	2 (8%) *	–	210 (170–240) *
Persiani (2020) [38]	69 vs 52 87 vs 34	TaTME	–	31 (45%)	–	– 294 (59)*	–	15 (25%)	–	– 259 (46)*
Bege (2010) [11]	50 vs 77	L-TME	–	26 (52%)*	5 (10%)	445 (117)	–	27 (35.1%) *	7 (9%)	414 (97)
Kayano (2011) [58]	50 vs 200	L-TME	–	14 (28%)	0	–	–	44 (22%)	0	–
Kuo (2013) [43]	17 vs 11	L-TME	–	–	3 (17.6%)	402 (210–570) *	–	–	1 (9.1%)	331 (210–450)
Morelli (2018) [46](Si)	19 vs 21	R-TME	–	7 (36.8%)	–	335 (64) *	–	7 (33.3%)	–	289 (42) *
Morelli (2018) [46](Xi)	19 vs 21	R-TME	–	4 (21.1%)	–	305 (51) *	–	6 (28.6%)	–	264 (39) *
Park (2014) [47]	44 vs 45	R-TME	–	5 (11.4%)	4 (9.1%)	230 (49) *	–	4 (8.9%)	2 (4.4%)	188 (53) *
Park (2014) [47]	41 vs 48	L-TME	–	8 (19.5%)	2 (4.9%)	242 (81) *	–	15 (31.3%)	4 (8.3%)	169 (53) *
Wang (2021) [51]	17 vs 23	R-TME	–	1 (5.9%)	1 (5.9%)	361 (41)*	–	2 (8.7%)	1 (4.3%)	324 (43) *
Wang (2021) [51]	34 vs 31	L-TME	–	2 (5.9%)	0 (0.0%)	338 (47) *	–	2 (6.5%)	0 (0.0%)	302 (53) *

*CRM* Circumferential resection margin 1 is composed outcome of CRM and DRM. * Significant difference between during and after learning curve. *L-TME* laparoscopic TME, *R-TME* robot-assisted TME, *TaTME* transanal TME

## Discussion

This systematic review aimed to provide an overview of the current literature regarding the learning curve of L-TME, R-TME and TaTME, and reveals the paucity of high-quality studies. The few available studies using a high-quality RA-CUSUM analysis based on intraoperative complications, postoperative complications or oncological outcomes show similar lengths of the learning curve for L-TME, R-TME, and TaTME. Additionally, although length of the learning curve is suggested to be similar, L-TME and TaTME might bear the risk of additional morbidity while obtaining the learning curve.

Only one L-TME study, three R-TME studies and three TaTME studies used the RA-CUSUM analysis based on clinically relevant outcomes such as intraoperative morbidity, postoperative morbidity or oncological outcomes [8, 9, 12, 14, 15, 35, 38]. Length of the learning curve was 50–70 for L-TME, 32–418 for R-TME and 36–54 for TaTME. This might suggest that the learning curve for R-TME is considerably longer than the learning curve of L-TME and TaTME. However, the results are influenced by the study of Lee et al., who found a learning curve of 177–418 procedures for R-TME [35]. As the authors state in their discussion, the substantial length of the learning curve might be due to the high number of examined cases: with increasing number of consecutive cases, length of the learning curve increases as well [5, 35, 62]. Taking this into account, the learning curve shows similar lengths between techniques: 50–70 procedures for L-TME, 32–75 procedures for R-TME and 36–54 procedures for TaTME [9, 11, 13–15]. This is in line with other systematic reviews evaluating the learning curve of minimal invasive techniques. A systematic review estimated the learning curve to be between 30 and 50 procedures in TaTME [16], and another systematic review estimated the learning curve of R-TME to be 37 procedures [17]. Furthermore, two systematic reviews compared length of the learning curve between L-TME and R-TME. One included studies with colorectal patients, both having benign and malign disease and reported a length between 5 and 310 for L-TME and 15–30 for R-TME [19]. A more recent systematic review only included studies with surgeons without laparoscopic experience and showed equal length of the learning curve: 44–55 for L-TME, and 41–55 for R-TME [20].

Although the length of the learning curve might not differ between the three techniques, L-TME and TaTME might bear the risk of additional morbidity while obtaining the learning curve. A L-TME and a TaTME study show higher rates of intraoperative and postoperative complications before reaching the learning curve, while no R-TME study shows a difference between these two phases [11, 18, 37]. Additionally, a systematic review comparing outcomes before and after the learning curve of TaTME showed less intraoperative complications, less anastomotic leakages and better quality of the TME specimen after the learning curve was obtained [16]. The evidence is scarce, but this might be in line with recently published data showing additional morbidity and higher local recurrence rates during the learning curve of TaTME [49, 50, 63–65]. This has also been suggested in a study assessing the learning curve of L-TME [10]. Perhaps the learning curve of L-TME and TaTME bear the risk of worsened oncological outcomes as these techniques differ significantly from the preceding ‘standard’ technique, while R-TME shows a high degree of similarity with the preceding L-TME technique. Subsequently, since most surgeons starting with R-TME have preceding experience with the L-TME technique, this influences the learning curve. While, on the other hand, surgeons starting with L-TME or TaTME start with a completely new technique, which might cause the additional morbidity during the learning curve.

The statements regarding length of the learning curve and additional morbidity during the learning curve should be interpreted cautious. Since, only limited amount of high-quality evidence exists, with lack of comparative studies, and a large amount of heterogeneity among studies. This is mainly caused by differences in patient-related factors, surgeon-related factors and statistical methods. First, regarding patient-related factors, inclusion- and exclusion criteria differ among studies, resulting in selection bias between studies. Furthermore, case-mix changes over the course of the learning curve: mostly an overrepresentation of “easy” patients is seen while climbing the learning curve, and more “difficult” patients are operated at the middle of the learning curve [14, 15]. Although case-mix can be controlled for by using a risk-adjusted analysis using the RA-CUSUM, this is only performed in a small number of studies.

Secondly, heterogeneity due to surgeon-related factors among studies exists as well: while some studies report on learning curves for individual surgeons, others report on institutional learning curves. As institutional learning curves might indicate the experience of the whole surgical team, they fail to address differences between individual surgeons. In addition, it is known that the first surgeon mastering the technique within an institution has a longer learning curve than the ones following, due to the institutional experience [66]. Furthermore, as experience with the minimal invasive technique and TME in general influences the learning curve, it is important to describe this. And although most studies reported the experience of the surgeon with the minimal invasive technique, details were lacking. Young surgeons who are at the start of their career, might have a longer learning curve than senior surgeons mastering minimal invasive surgery since the latter might have experience in performing open or L-TME [67]. Additionally, as R-TME and TaTME have been introduced 10–15 years later than L-TME, most studies addressing the learning curve of R-TME and TaTME included surgeons who already had experience with L-TME. This might be an important confounder while assessing the learning curve of L-TME with R-TME or TaTME, but it is inherent to the clinical practice. Finally, since TaTME is generally not used for an abdominoperineal resection, while this is performed using L-TME or R-TME, differences regarding the indication of the technique complicate the comparability of these techniques.

Thirdly, regarding heterogeneity among studies caused by the used statistical analyses, differences could be due to the used outcome measure to establish the learning curve, the used statistical technique and the used cut-off point. Regarding the used outcome measure to establish the learning curve, operative time is often used for the learning curve. However the outcome is said to be a poor surrogate for clinical outcomes, and mere a reflection of efficiency [5, 68]. Instead, clinical outcomes that are of interest for patients should be used to assess the learning curve [5, 68]. For example, intraoperative complications, major postoperative complications [69], positive CRM rate and for the long term local recurrence rate [70]. Additionally, in order to provide comparable outcomes, clear definitions according to international standards should be used [71].

Regarding the used statistical technique, several methods for the analyses are used: split group analysis (SGA), moving average analysis (MAA), CUSUM and RA-CUSUM. For SGA, patients are arbitrarily divided into two or more groups, based on the chronological order. Since these learning curves are dependent on how groups were divided, it could be doubted whether SGA is suitable for analyzing learning curves [5, 28, 39, 41]. MAA learning curves are based on operative time alone. As operative time might not be an adequate indicator of proficiency, this technique might not be suitable either [72]. CUSUM and RA-CUSUM analyses are more complex methods used to continuously monitor outcomes. The CUSUM is a chronically ordered cumulative sum of the difference between the outcome of the procedure and the average of the studied cohort or a predefined cut-off point based on literature [14, 15]. The RA-CUSUM analysis is the more sophisticated method, correcting for case-mix that may influence the risk of an event [14, 15, 35, 73]. However, both methods have been developed to monitor processes known to be adequate, while signaling inadequacy. For surgeons carrying out a procedure they have not yet performed regularly the learning curve CUSUM (LC-CUSUM) might be more suitable. This analysis assumes inadequacy of the surgeon, while signaling adequacy [62]. This method could be used when the surgeon has no experience with the procedure, as is the case with young surgeons starting with L-TME or R-TME. Or it can be used for describing the learning curve of an experienced colorectal surgeon starting with TaTME, since this procedure is to a large extent different from the “top-down” approach used in open, L-TME and R-TME.

Finally, regarding the used cut-off points, all CUSUM methods can be performed using limits based on averages of the cohort or using literature-based limits. Using averages of the cohort complicates comparison with other studies. And, as mentioned earlier, using averages causes the length of the learning curve to increase with larger cohort size [5, 62]. Therefore, literature-based limits are preferred. Furthermore, the point at which ‘proficiency’ is reached influences the length of the learning curve as well. Studies used two different points to identify proficiency: the point at which the graph deflects or the point at which a stabilization occurs. Both methods are used, while different outcomes are produced [13, 24, 52]. Therefore, it has been proposed that a learning plateau (i.e., stabilization) should reach a predefined competency level, based on estimates available in literature [5]. As not all studies included in this systematic review provided the point of stabilization, while the point of deflection was provided in every study, this was used in our analysis for assessing the length of the learning curve.

Although this is the first systematic review to provide an overview of the literature regarding the learning curves of all three minimal invasive techniques of TME and the methods used to establish them, it cannot draw a definite conclusion regarding differences in length of the learning curves and differences in additional morbidity during the learning curve of L-TME, R-TME and TaTME. Clearly, more high-quality studies are necessary to shed light on the learning curve of minimally invasive techniques for rectal resections. We suggest that this should preferably be performed with comparative studies, while controlling for patient-related factors (i.e., risk-adjusted analysis), and surgeon-related factors such as experience with TME in general and experience with the specific minimal invasive technique. In addition, if former experience with the TME procedure is limited (i.e., beginning surgeon or adhering to a new technique like TaTME) the LC-CUSUM should be used. Furthermore, we propose that learning curves should be established for individual surgeons, based on the following clinically relevant outcome variables: intraoperative morbidity, (major) postoperative morbidity and positive CRM. Additionally, clear outcome definitions should be reported and learning curves should be estimated using literature-based limits. Finally, comparison of outcomes during and after the learning curve should be performed, to investigate whether the learning curve is associated with additional morbidity.

## Data Availability

Data is available on request, this includes template data collection forms, data extracted from included studies, data used for analyses and any other materials used in the review.
